# Using Personal Values to Understand the Motivational Basis of Amity Goal Orientation

**DOI:** 10.3389/fpsyg.2018.02736

**Published:** 2019-01-09

**Authors:** Liat Levontin, Anat Bardi

**Affiliations:** ^1^Faculty of Industrial Engineering and Management, Technion – Israel Institute of Technology, Haifa, Israel; ^2^Department of Psychology, Royal Holloway University of London, Egham, United Kingdom

**Keywords:** values, goal orientations, achievement goals, amity goal orientation, motivation

## Abstract

Values are broad motivations that can serve as the basis for goals. We propose that values can be used to understand the motivational basis of amity goal orientation, a prosocial goal orientation within achievement situations. We offer theory and empirical evidence relating personal values to amity goal orientation and other achievement goal orientations. Specifically, the results of three studies and a mini meta-analysis suggest that the prosocial value of benevolence is positively related to amity goal orientation and can be interpreted as the motivational basis of amity goal orientation. Furthermore, power values are positively related to performance-approach goal orientation; self-direction values are positively related to mastery goal orientation, and security values are positively related to performance-avoidance goal orientation. These findings can explain the pattern of correlations previously found among achievement goal orientations, and open up the potential for new research on amity goal orientation as well as other value-based achievement goal orientations.

## Introduction and Overview

The topic of achievement goal orientations has received a great deal of research attention in the last few decades, with meta-analyses covering over 300 studies of the antecedents and consequences of achievement goal orientations (Payne et al., 2007; Hulleman et al., 2010; Huang, 2011). Goal orientation research has led to both theoretical advancements and useful advice to practitioners, such as educators and managers, in the quest to better understand achievement motivation and its influence on performance in achievement situations (DeShon and Gillespie, 2005; Kaplan and Maehr, 2007; Payne et al., 2007).

Recent research (Levontin and Bardi, 2018) introduced a new goal orientation, amity goal orientation, showing its important consequences for success. However, the motivational basis of this new achievement goal orientation remains unclear. In this paper, we suggest that a fruitful way of understanding the motivational basis of amity goal orientation, and other achievement goal orientations, is using values, as values are broad motivational goals that are likely to underlie contextual goals. As the circumplex structure of values (Schwartz, 1992) is well established, it can serve to understand the motivations underlying goal orientations. Moreover, the value circumplex covers a comprehensive set of value-related motivations that are likely to underlie many different goals. We propose that amity goal orientation is associated, and probably based on benevolence values.

## Achievement Goal Orientations

Goal orientation theory is focused on goals that are pursued or perceived by individuals in achievement situations resulting in different patterns of cognition, affect, and behavior (Nicholls, 1984; Dweck and Leggett, 1988). The original theory portrays two goal orientations. The first goal orientation is to develop one’s competence by acquiring new skills and developing mastery in new situations (labeled as task goal, learning goal, or *mastery* goal). The second goal orientation is the goal of demonstrating competence or avoiding exhibiting a lack of competence by seeking favorable judgments from others and avoiding negative ones (labeled as ability goal, ego goal, or *performance* goal).

Each of the goal orientations leads to different preferences within achievement situations. Individuals with a high mastery goal orientation are willing to risk erring in order to learn. They also prefer to engage in new, demanding, and challenging tasks rather than repeat familiar ones. Conversely, individuals with a high-performance goal orientation tend to abstain from learning opportunities in which there is a risk of erring. They, therefore, favor performing familiar tasks in which they feel safe, so as not to make any errors, and are reluctant to opt for difficult tasks (Elliott and Dweck, 1988). Mastery and performance goal orientations also differ in their emotional consequences. Mastery goal orientation elicits enjoyment, optimism, and intrinsic interest (Dweck, 1986; Butler, 1987; Dweck and Leggett, 1988), whereas performance goal orientation elicits helplessness, negative affect, anxiety, and stress (Dweck and Leggett, 1988).

This model has been expanded later to include factors within the basic division, of which one of the most influential has been the inclusion of an orthogonal factor of approach (performance – aiming for high performance; mastery–aiming to learn) and avoidance (performance – avoiding poor performance; mastery – avoiding loss of knowledge and skills; Elliot and Church, 1997; VandeWalle, 1997; Midgley et al., 1998; Elliot, 1999; Elliot and McGregor, 2001; Elliot et al., 2011).

Recent research introduced amity achievement goal orientation, a pro-social goal orientation, the goal to improve others’ competence together with one’s own competence, which involves cooperation and assisting others to succeed (Levontin and Bardi, 2018). It was found that best performance results, both in education and work settings, are achieved by mastery combined with amity goal orientations.

## The Schwartz (1992) Value Theory

Values (e.g., security and self-direction) convey broad life-goals that are important to us in our lives (Schwartz, 1992). They serve as guiding principles in people’s lives and are ordered according to their importance which varies across individuals (Rokeach, 1973; Schwartz, 1992). Their broad nature leads them to have an over-arching effect across contexts. For example, a person who values benevolence is likely to be motivated by benevolence values at home, at work, and with friends. Values are quite stable (e.g., Goodwin et al., 2012; Vecchione et al., 2018), and as such function as ongoing motivators of perceptions, attitudes, goals, and behaviors (Schwartz, 2011).

**FIGURE 1:**
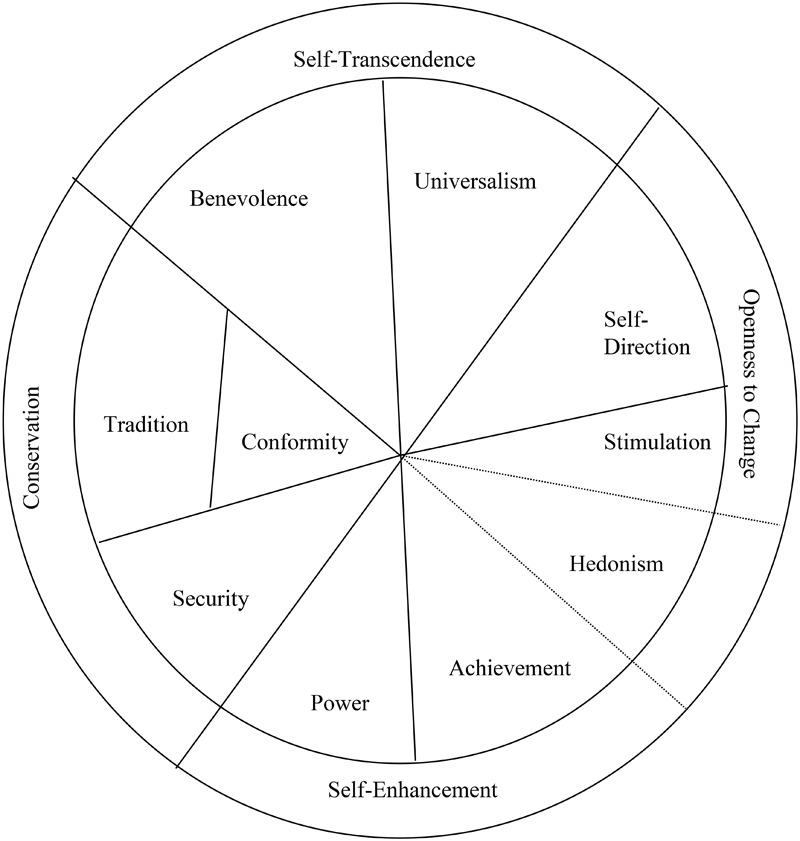
The structure of values (Schwartz, 1992).

Schwartz (1992) presented a comprehensive model of values which defines 10 broad values that form a circle according to the motivation that underlies each of them (see Figure 1). Every two adjacent values in the circle share an underlying motivation. For example, benevolence and universalism share a pro-social motivation and can often be pursued simultaneously with the same pro-social goal or act. In contrast, values that emanate from opposite sides of the center of the circle are based on conflicting motivations, as a pursuit of one often hampers the pursuit of the other (e.g., conformity and self-direction). The circle of values is based on two bi-polar orthogonal dimensions. The first dimension is of openness to change versus conservation. It arrays values according to the extent to which they motivate people to be open to new ideas and experiences (openness to change, including self-direction, stimulation, and sometimes hedonism) versus the motivation to preserve the status quo and the certainty it provides (conservation, including security, conformity, and tradition). The second dimension is self-enhancement versus self-transcendence. It arrays values according to the extent to which they motivate people to enhance personal interests even at the expense of others (self-enhancement, including power, achievement, and sometimes hedonism) versus the motivation to promote the welfare of others (self-transcendence, including benevolence and universalism). The 10 values and their structure of relations have been established in cross-cultural research in more than 70 countries from around the world (Schwartz, 2011).

As the value circle is based on a motivational continuum, Schwartz (1992) and Schwartz and Boehnke (2004) advocate dividing the circle to more specific or to broader areas when a different division better represents hypotheses. Accordingly, in the current research, we use the division of universalism to the three parts that Schwartz (1992) suggests (see also Lee et al., 2008; Bardi et al., 2009). *Universalism-social* expresses a general prosocial motivation and societal concern and includes the specific values of equality, social justice, and a world at peace (Schwartz and Boehnke, 2004; Schwartz, 2007). Schwartz (1992) noted that these values are the closest to benevolence values in their spatial location due to this shared pro-social motivation. *Universalism-intellect* expresses an open-minded intellectual motivation and includes the specific values of broad-mindedness and wisdom. Schwartz (1992) noted that these two values are the closest to self-direction values in their spatial representation, probably due to the motivation of independent judgment that they share with self-direction values. Finally, *universalism-nature* expresses the motivation to protect nature and includes the specific values of protecting the environment, a world of beauty, and unity with nature (Schwartz and Boehnke, 2004).

## The Expected Relations Between Values, Amity Goal Orientation, and Other Achievement Goal Orientations

Schwartz (2011) has suggested that the circle of values represents a structure of basic motivations. Similarly, motivated action theory (DeShon and Gillespie, 2005) interprets goal orientations as representations of values (among other motivational variables like needs and drives). Thus, we suggest that the basic values that guide people’s lives across contexts are also likely to be represented in goal orientations in achievement contexts and lead to a tendency for goal orientations that express the person’s important values. We next consider the broad value contents and theorize about achievement goal orientations that express them. We start with the values that are likely to motivate amity goal orientation, and we progress to cover the rest of the circle to hypothesize on achievement goal orientations that are motivated by the other values.

### Self-Transcendence

We suggest that amity goal orientation is based on the social part of self-transcendence. Self-transcendence values express the motivation for transcending beyond one’s selfish interests and promoting the welfare of all people and nature. Promoting nature does not apply to most achievement situations. Another part of universalism, universalism-intellect, also does not seem to relate particularly to the prosocial goal of amity. This leaves us with the social aspect of self-transcendence values, which expresses the motivation for being prosocial. *Amity achievement goal orientation* (Levontin and Bardi, 2018) is focused exactly on such goals. People who hold amity goal orientation aim at the improvement of others’ competence together with their competence and aim for cooperation and assisting others to succeed. Because this goal orientation operates in relationships with concrete people, it should be more directly based on benevolence values than on universalism-social values as the latter are focused on being prosocial toward generalized populations (as in the value of social justice and world at peace) and outgroups. Hence, this goal orientation is likely to be associated mainly with benevolence and sometimes also with universalism-social values.

### Self-Enhancement

These values express the motivation to enhance selfish interests of success according to social standards and of prestige, even at the expense of others. This higher order value is therefore likely to motivate achievement goal orientations in which one strives for external rewards such as salary or bonuses or other clear indicators of success, such as managers’ evaluations, promotions, and school grades. One of the most studied achievement goal orientations is focused exactly on such goals, namely, *performance-approach goal orientation* (e.g., Midgley et al., 2001). People who hold performance-approach goal orientation aim to demonstrate their ability and competence to others and to demonstrate higher competence compared to others. Hence, we hypothesize that performance-approach goal orientation will be associated with self-enhancement values, and particularly with power values as they express the wish for prestige. Achievement values could also be sometimes associated with performance-approach goal orientation as they express the wish for success through social standards. Hedonism values, which are also often part of self-enhancement values, seem less relevant to achievement situations, although they are compatible with performance-approach goal orientation as monetary rewards lead to the ability to enjoy luxuries. Indeed self-enhancement values were found to predict performance-approach goal orientation (Pulfrey and Butera, 2013).

### Openness to Change

These values express the motivation for novelty and challenge. Hence, they are likely to lead to achievement goals that are aimed at mastering challenges. A much-studied achievement goal orientation that focuses on mastering challenges is *mastery (approach) goal orientation* (e.g., Elliot and McGregor, 2001). People who hold mastery goal orientation focus on learning and skill development. Hence, we hypothesize that mastery goal orientation will be associated with openness to change values, and mainly with self-direction values that are focused on intellectual novelty. Stimulation values are focused on stimulating activities and therefore are less relevant to most achievement situations. Another type of value that could sometimes be related to mastery goal orientation is universalism-intellect values that express an open-minded intellectual motivation.^1^

### Conservation

These values express the motivation for maintaining things as they are, including the motivation for predictability and for avoiding risks. This motivation is likely to be related to achievement goal orientations that are focused on avoiding risks, such as the risk of failure in embarking on a new task, and risk of failure in general. A much-studied achievement goal orientation that focuses on avoiding failure is *performance-avoidance goal orientation* (e.g., Elliot and Church, 1997*).* People who hold a performance-avoidance goal orientation focus on avoiding negative outcomes such as showing lack of ability, or performing poorly compared with others. Hence, individuals with performance-avoidance goal orientation are likely to be motivated to be safe, which reflects conservation values. We therefore hypothesize that performance-avoidance goal orientations will be associated with conservation values, and particularly with security values. Tradition and conformity values may not be direct motivators of performance-avoidance goal orientations, but keeping to traditional ways of working and conforming to superiors and rules and regulations may support the general aim of those who hold performance-avoidance goal orientation in avoiding the risk of negative judgments in achievement situations.

## The Current Research

Studies 1 and 2 examine the pattern of relations between values and achievement goal orientations in an academic achievement context. Study 3 examines whether the findings of studies 1 and 2 can be extended to another achievement context (work). All studies were conducted in accordance with APA ethical guidelines and approved by the university’s ethics committee in adherence to the highest ethical standards providing full anonymity to all participants.

Although values are likely to motivate goal orientations and hence to be correlated with them, we do not expect strong correlations between broad values and contextualized goals, such as achievement goal orientations. As explained by Maio (2010), theoretically derived links between values and other variables are not likely to be strong because the broad nature of values means that the same value can be expressed through more than one goal, depending on the person’s interpretation of situations. The advantage of values is their circumplex structure, which enables hypothesizing and testing relations to the system of values as a whole. In three studies, we examined the hypothesized associations as well as the joint spatial relations of four achievement goal orientations, with the broad values of the Schwartz (1992) value theory, splitting universalism into three sub-types (universalism-intellect, universalism-social, and universalism-nature). By examining the joint structures of values and achievement goal orientations, we can also gain insights regarding the potential connections among achievement goal orientations. For example, if we indeed find that mastery goal orientation and performance-avoidance goal orientation are associated with conflicting values, this would also mean that these two goal orientations are not likely to co-occur. We expect the following:

1. Amity goal orientation is expected to be associated with benevolence values.
2. Performance-approach goal orientation is expected to be associated with power values.
3. Mastery goal orientation is expected to be associated with self-direction values.
4. Performance-avoidance goal orientation is expected to be associated with security values.

We used correlations and a mini meta-analysis to test these hypotheses.

**FIGURE 2:**
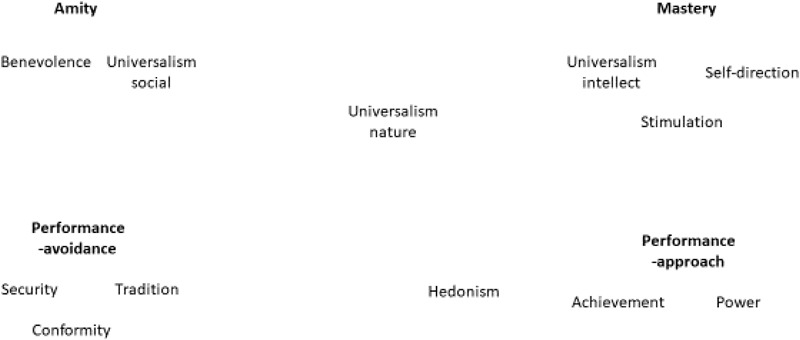
The expected spatial pattern of relations between goal orientations and values.

5. The hypothesized spatial pattern of relation between goal orientations and values in a two-dimensional space is presented in Figure 2. We used factor analysis and MDS to test the structure hypothesis.

In all three studies, we report all measures included in the study and all data collected are included.

## Study 1

This study was conducted in the context of achieving at a university. It therefore included university students and asked about achievement in one’s studies.

### Methods

#### Participants and Procedure

One hundred and forty-six first-year business administration students from Israel (*M*_age_ = 22.47, *SD* = 2.05, 44.8% females) participated in this study in return for course credit. The sample size of all three studies was determined by considering both the analysis of the circumplex structure of the values and by considering the size needed for the correlations between values and achievement goal orientations. The structure of the value circumplex has repeatedly been found with samples of just over 100 participants (see, e.g., Borg et al., 2017). All our hypotheses were directional, and we expected correlations in the order of 0.25 (see Maio, 2010, reviewed above) and therefore for an 80% statistical power, around 130 participants are needed. Participants reported their values and their goal orientations.

#### Instruments

##### Values

Values were measured with the 44-item version of the Schwartz Value Survey (SVS, Schwartz, 1992). This version includes all the items found to have similar meanings across cultures (Gandal et al., 2005). Participants rated the importance of each value item “as a guiding principle in my life” on a nine-point scale, ranging from -1 (opposed to my principles) via 0 (not important) to 7 (of supreme importance). The asymmetry of the scale reflects the natural distribution of distinctions that individuals make when thinking about the importance of values (Schwartz and Bardi, 2001). Schwartz (1992) recommends controlling for individual differences in scale use, and indeed in a recent meta-analysis, this has been found to result in stronger and more meaningful findings (Parks-Leduc et al., 2015). In addition to the statistical advantage of controlling for scale use, this procedure is also more theoretically appropriate. The reason for this is that values operate in a system (see Rokeach, 1973); hence, in each situation, more than one value is relevant. For example, if we are asked to comply with our boss’s unreasonable request, we may pursue conformity and security values by complying or pursue self-direction values by not complying (see Bardi et al., 2009). Hence, in any given situation what is most important is how much we prioritize a value compared to all our other values. This is portrayed most directly when the values scores are subtracted from the person’s mean rating of all the values. Therefore, we computed for each person the mean importance rating across all 44 items and subtracted that mean from the response to each item, such that each item score reflects the deviation of that item from the person’s mean response. For further information about the reliability and validity of this measure, see Schwartz (2005). Means and scale reliabilities are presented in Table 1. Reliabilities were similar to those found in previous research (see Schwartz, 2005).

**Table 1:** Values and goal orientations scales’ characteristics.

Type	Study 1				Study 2				Study 3			
*Values*	No of items	α	*M*	*SD*	No of items	α	*M*	*SD*	No of items	α	*M*	*SD*
Power	3	0.58	3.54	1.24	3	0.63	3.59	1.29	3	0.68	3.04	1.67
Achievement	4	0.67	4.89	0.91	4	0.68	4.96	0.87	4	0.77	4.76	1.33
Hedonism	2	0.70	4.29	1.32	2	0.69	4.47	1.30	2	0.74	4.27	1.51
Stimulation	3	0.77	4.00	1.28	3	0.76	4.13	1.31	3	0.80	3.81	1.72
Self-direction	5	0.60	4.87	0.86	5	0.56	4.93	0.76	5	0.62	5.25	0.99
Universalism-intellect	2	0.20	5.15	0.91	2	0.30	5.20	0.90	2	0.43	5.16	1.29
Universalism-nature	3	0.70	2.98	1.27	3	0.72	3.11	1.32	3	0.81	3.62	1.73
Universalism-social	3	0.71	4.64	1.28	3	0.71	4.64	1.29	3	0.70	5.05	1.44
Benevolence	5	0.72	4.94	0.88	5	0.68	4.97	0.83	5	0.74	5.16	1.14
Tradition	5	0.63	3.18	1.23	5	0.66	3.21	1.25	5	0.70	3.41	1.54
Conformity	4	0.63	4.45	0.93	4	0.61	4.39	0.94	4	0.72	4.46	1.37
Security	5	0.58	4.64	0.86	5	0.58	4.75	0.87	5	0.67	4.76	1.15
**Achievement goals**												
Performance-avoidance	3	0.67	3.49	1.23	3	0.71	3.46	1.29	4	0.86	4.07	1.40
Mastery	3	0.80	5.78	0.94	3	0.80	5.46	1.06	5	0.87	5.88	0.99
Performance-approach	3	0.83	4.29	1.38	3	0.85	4.23	1.43	4	0.86	5.59	1.17
Amity	4	0.78	4.69	1.30	4	0.81	4.77	1.13	8	0.83	5.32	1.00

##### Achievement goal orientations

Three of the goal orientations were measured using the items of one of the most frequently used academically related goal orientations questionnaire (Elliot and McGregor, 2001). Amity goal orientation was measured using four items that measure the motivation for cooperation with others in an academic achievement situation, the willingness to help others to succeed, and the willingness to develop and improve non-competitive relations with others (Levontin and Bardi, 2018, see items in Appendix 1). Participants indicated on a scale from 1 (*not at all true of me*) to 7 (*very much true of me*) the extent to which they are motivated by performance-approach (three items, α = 0.83, e.g., “It is important for me to do better than other students”), mastery (three items, α = 0.80, e.g., “I desire to completely master the material presented in this class”), performance-avoidance (three items, α = 0.69, e.g., “My goal for this class is to avoid performing poorly”), and amity (four items, α = 0.78, e.g., “It is important to me that my friends will succeed as well as I do”) goal orientations in the current class (see Table 1). For comparability between values and goal orientations, we computed for each person the mean importance rating across all goal orientation items and subtracted that mean from the response to each item, such that each item score reflects the deviation of that item from the person’s mean response. This was particularly important for the analysis of the joint structure of values and goal orientations, as for this analysis, it is important how dominant each value and goal orientation is compared to other values and goal orientations.

### Results and Discussion

Our results show that amity goal orientation is important to students (*M* = 4.69, *SD* = 1.13), more than performance-approach [*M* = 4.29, *SD* = 1.38, *t*_(145)_ = 2.69, *p* = 0.008] and performance-avoidance goal orientations [*M* = 3.49, *SD* = 1.23, *t*_(145)_ = 8.55, *p* < 0.001], second only to mastery goal orientation [*M* = 5.78, *SD* = 0.94, *t*_(145)_ = -9.96, *p* < 0.001].

The correlations between goal orientations and values are presented in Table 2. Since we tested four hypotheses, a correlation with a p value smaller than 0.0063 would support the hypotheses, correcting for possible alpha error inflation. As expected, amity goal orientation is positively related to benevolence values (*r* = 0.24, *p* = 0.004), and performance-approach goal orientation is positively related to power values (*r* = 0.27, *p* = 0.001). The correlation between mastery goal orientation and self-direction values is in the expected direction (*r* = 0.22, *p* = 0.008). We also expected that performance-avoidance goal orientation would be positively related to security values, but this correlation was not significant (*r* = 0.16, *p* = 0.052).

**Table 2:** Correlations between values and goal orientations.

	Study 1				Study 2				Study 3			
	Amity	Performance approach	Mastery	Performance avoidance	Amity	Performance approach	Mastery	Performance avoidance	Amity	Performance approach	Mastery	Performance avoidance
Power	-0.22**	**0.27^∗∗^**	-0.09	0.23**	-0.26**	**0.29^∗∗^**	-0.11*	0.24**	-0.15*	**0.15^+^**	0.02	0.03
Achievement	-0.15^+^	0.13	0.21*	-0.03	-0.16**	0.24**	0.05	0.03	-0.12	0.05	0.28**	-0.16*
Hedonism	-0.07	0.05	-0.09	0.13	-0.15**	0.13*	-0.18**	0.11*	-0.11	0.16*	-0.15*	0.13^+^
Stimulation	0.03	-0.08	0.01	-0.07	-0.05	0.02	-0.09^+^	-0.04	-0.11	0.04	0.22**	-0.11
Self-direction	-0.08	-0.01	**0.22^∗∗^**	-0.01	-0.13*	0.09^+^	**0.13^∗^**	-0.03	-0.26**	0.11	**0.26^∗∗^**	-0.04
Universalism-intellect	-0.07	0.04	-0.00	-0.12	-0.07	0.02	0.06	-0.13*	0.02	-0.13^+^	0.25	-0.15*
Universalism-nature	0.12	0.01	0.01	-0.07	0.12*	-0.02	0.03	-0.13*	0.13^+^	-0.24**	0.11	-0.05
Universalism-social	0.28**	-0.12	-0.20*	-0.09	0.24**	-0.16**	-0.02	-0.11*	0.14^+^	-0.11	-0.11	0.03
Benevolence	**0.24^∗∗^**	-0.20*	0.010	-0.11	**0.30^∗∗^**	-0.27**	0.13*	-0.22**	**0.19^∗∗^**	-0.17*	0.08	-0.15*
Tradition	0.09	-0.07	-0.04	-0.06	0.13*	-0.18**	0.01	-0.01	0.17*	-0.20**	-0.19*	0.15*
Conformity	-0.14	‘0.01	-0.03	0.15^+^	-0.02	-0.07	0.10^+^	0.09^+^	0.13^+^	-0.06	-0.05	-0.04
Security	-0.17	0.05	-0.06	**0.16^+^**	-0.10^+^	0.06	-0.08	**0.16^∗∗^**	0.01	0.15*	-0.18*	0.04

*^∗∗^*p* <0 .01; ^∗^*p* < 0.05; ^+^*p* < 0.10. Hypothesized correlations are in bold.*

**FIGURE 3:**
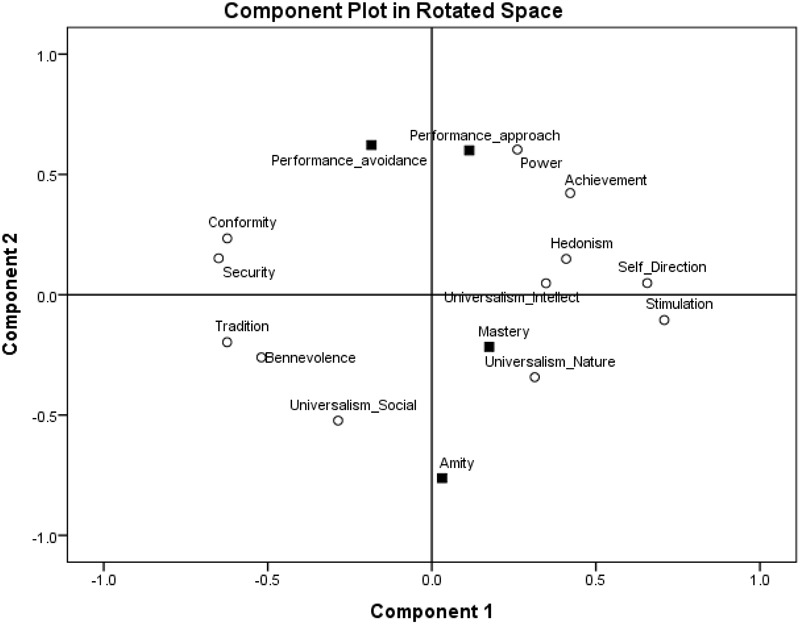
Component loadings of goal orientations and values in a two-dimensional space, Study 1.

To find where these goal orientations are located in the two-dimensional values space, we ran a principal component analysis on the four goal orientations and the 12 values with Varimax rotation, limiting the number of factors to two, in line with the circumplex structure of the values (see Bardi et al., 2009). The Varimax-rotated component loadings are presented in Figure 3. In examining the structure hypothesis, we look at the general areas in which goal orientation emerge, rather than specific proximities to specific values, due to random error in the representation of variables that stem from different measures in a two-dimensional space. As expected, amity goal orientation emerged in the area of self-transcendence values close to universalism-social and benevolence values. Performance-approach goal orientation emerged in the area of self-enhancement values close to power and achievement values. Mastery goal orientation emerged in the area of openness values close to self-direction, universalism-intellect, and stimulation values. Finally, performance-avoidance goal orientation emerged in the area of conservation values close to security and conformity values. Importantly, the circular structure of values was kept when goal orientations were added to the analyses which suggest that the underlying motivations behind the values may also be relevant to goal orientations.

Probably due to the relations not only between variables that measure similar motivations but also between types of measures (values and goal orientations), achievement goal orientations have formed a dimension of goal orientations with positive loadings of performance-avoidance goal orientation (0.622) and performance-approach goal orientation (0.600) on that dimension and negative loadings of mastery goal orientation (-0.217) and amity goal orientation (-0.762) on that dimension.

**FIGURE 4:**
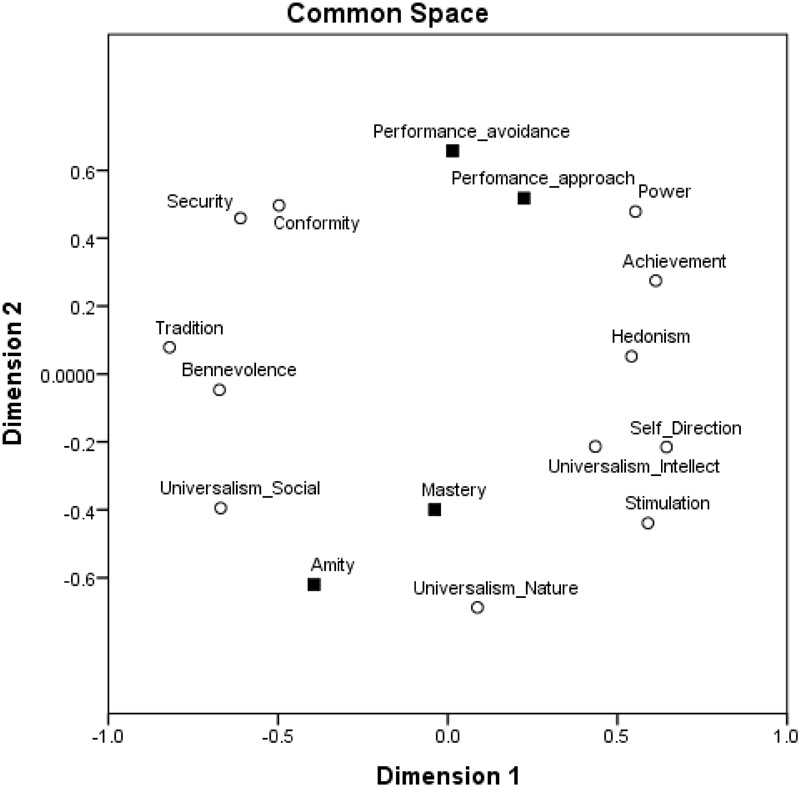
MDS of achievement goal orientations and values in a two-dimensional space, Study 1.

To further asses the structure of relations among goal orientations and values and their locations around the circular motivational continuum, we followed a confirmatory procedure (Schwartz et al., 2012) and ran multidimensional scaling (MDS) analyses (Borg and Lingoes, 2012). We assessed whether the 12 values and four goal orientations form structures comparable to the structure obtained by the exploratory analysis using MDS. We used the SPSS MDS Proxscal analysis with Euclidian distance measures and the inter-correlations among the centered variables as the data. We specified an ordinal MDS, with the primary approach to ties and Torgerson initial configuration. Results are presented in Figure 4. The similarity of the results to those obtained by exploratory factor analysis is striking. Furthermore, this result is a good representation of the data: Normalized raw stress = 0.031, Stress-I = 0.177, Stress-II = 0.460, S-Stress = 0.061, DAF (dispersion accounted for) = 0.969, Tucker’s coefficient of congruence = 0.984. Specifically, as expected, amity goal orientation emerged in the area of self-transcendence values close to universalism-social and benevolence values, performance-approach goal orientation emerged in the area of self-enhancement values close to power and achievement values, mastery goal orientation emerged in the area of openness values close to universalism-intellect, stimulation and self-direction values, and performance-avoidance goal orientation emerged in the area of conservation values close to conformity and security values.

The results of this study suggest that amity goal orientation is conceptually related to the self-transcendence values of benevolence and universalism-social. The results also extend the achievement goal orientations approach to include personal values as possible antecedents of other goal orientations. In this study, we measured academic achievement goal orientations of first-year business students. In the next study, we sought to see if these findings generalize to students of all years and more majors of undergraduate studies.

## Study 2

This study, as Study 1, was done in the context of achieving at a university. It therefore included university students and asked about achievement in one’s studies.

### Methods

#### Participants and Procedure

Three hundred and fifty-two students from Israel (Mage = 22.93, *SD* = 2.16, 43.7% females) participated in this study in return for course credit. 33.7% (*N* = 119) were business administration students, 27.2% (*N* = 96) were accountant students, 24.6% (*N* = 87) were economics students, and the other 31.7% were students from all over campus including law (*N* = 15), psychology (*N* = 14), and communication (*N* = 8) students. Data were collected for a few weeks during which any student who wished to participate was welcome. Participants reported their values and their goal orientations using the same instruments as in Study 1. Means and scale reliabilities are presented in Table 1 and were largely similar to findings of previous research (see Schwartz, 2005).

### Results and Discussion

Replicating Study 1, amity goal orientation was important to students (*M* = 4.77, *SD* = 1.13), more than performance-approach [*M* = 4.23, *SD* = 1.43, *t*_(351)_ = 5.42, *p* < 0.001] and performance-avoidance goal orientations [*M* = 3.46, *SD* = 1.29, *t*_(351)_ = 14.49, *p* < 0.001], second only to mastery goal orientation [*M* = 5.46, *SD* = 1.06, *t*_(351)_ = -9.17, *p* < 0.001].

The correlations between goal orientations and values are presented in Table 2. As in Study 1, a correlation with a p value smaller than 0.0063 would support the hypotheses, correcting for possible alpha error inflation. As expected, and replicating the results of Study 1, amity goal orientation is positively related to benevolence values (*r* = 0.30, *p* < 0.001), performance-approach goal orientation is positively related to power values (*r* = 0.29, *p* < 0.001), and performance-avoidance goal orientation is positively related to security values (*r* = 0.16, *p* = 0.003). The correlation between mastery goal orientation and self-direction values is in the expected direction (*r* = 0.13, *p* = 0.018).

**FIGURE 5:**
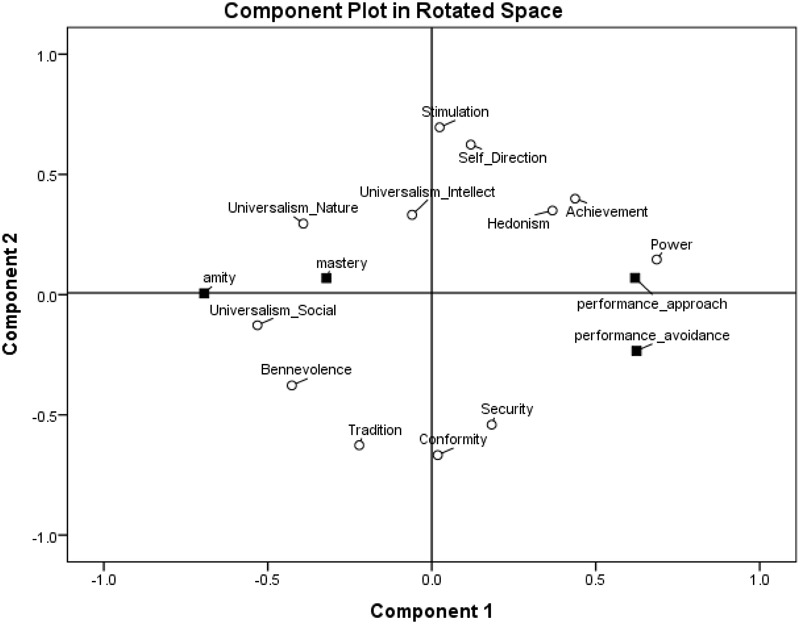
Component loadings of goal orientations and values in a two-dimensional space, Study 2.

To find where these goal orientations are located in the two-dimensional values space, we ran a principal component analysis on the four goal orientations and the 12 values. The Varimax-rotated component loadings are presented in Figure 5. As expected, amity goal orientation emerged in the area of self-transcendence values close to universalism-social and benevolence values. Performance-approach goal orientation emerged in the area of self-enhancement values close to power and achievement values. Mastery goal orientation emerged in the area of openness values close to universalism-intellect values. Finally, performance-avoidance goal orientation emerged in the area of conservation values close to security and conformity values. Importantly, as in Study 1, the circular structure of values was kept when goal orientations were added to the analyses in support of the suggestion that the underlying motivations behind values may also be relevant to goal orientations.

**FIGURE 6:**
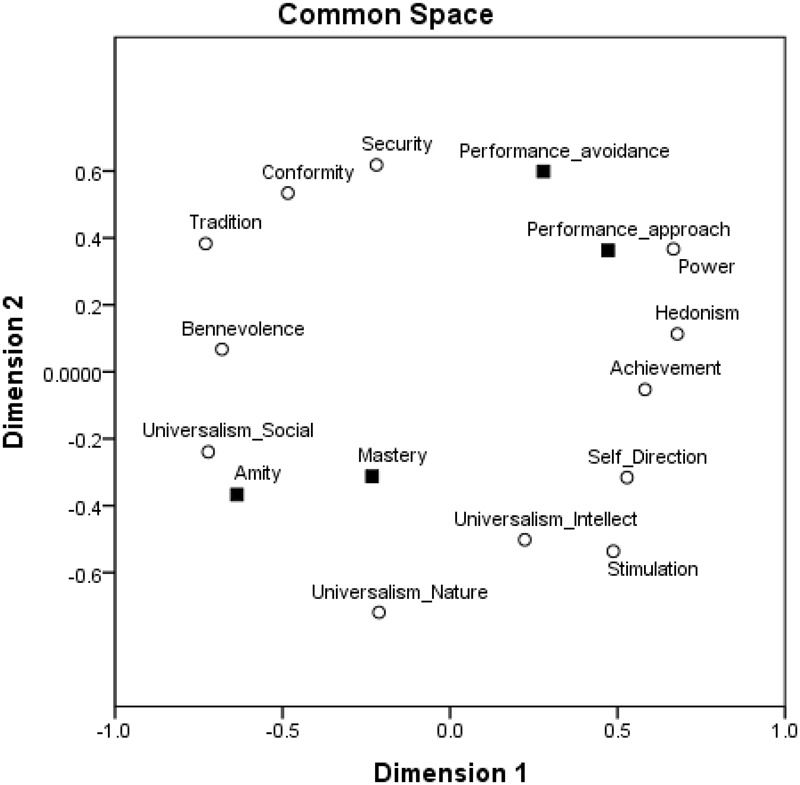
MDS of achievement goal orientations and values in a two-dimensional space, Study 2.

To further asses the structure of relations among goal orientations and values and their locations around the circular motivational continuum, we followed a confirmatory procedure and ran MDS analyses as in Study 1. We assessed whether the 12 values and four goal orientations form structures comparable to the structure obtained by the exploratory analysis using MDS. We used the SPSS MDS Proxscal analysis with Euclidian distance measures and the inter-correlations among the centered variables as the data. We specified an ordinal MDS and Torgerson initial configuration. Results are presented in Figure 6. The similarity of the results to those obtained by exploratory factor analysis is once again striking. Furthermore, this result is a good representation of the data: Normalized raw stress = 0.024, Stress-I = 0.156, Stress-II = 0.404, S-Stress = 0.050, DAF (dispersion accounted for) = 0.976, Tucker’s coefficient of congruence = 0.988. Specifically, as expected, amity goal orientation emerged in the area of self-transcendence values close to universalism-social and benevolence values; performance-approach goal orientation emerged in the area of self-enhancement values close to power, hedonism, and achievement values; mastery goal orientation emerged in the area of openness values close to universalism-intellect, stimulation, and self-direction values; and performance-avoidance goal orientation emerged in the area of conservation values close to security, conformity, and tradition values.

The results of Studies 1 and 2 suggest that in academic achievement situations, in support of H1, amity goal orientation is associated with benevolence values. H2 was also supported since performance-approach goal orientation was associated with power values in both samples. H3 received support as mastery goal orientation was associated with self-direction values in both samples. H4 was almost fully supported as performance-avoidance goal orientation was significantly associated with security values in Study 2 and marginally in Study 1.

The results of this study’s multidimensional analyses suggest that amity goal orientation is conceptually related to the self-transcendence values of benevolence and universalism-social. The results also extend the achievement goal orientations approach to include personal values as possible antecedents of other goal orientations. In Studies 1 and 2, we measured academic achievement goal orientations. It is not clear if values are meaningfully related to work goal orientations as well. We tested this possibility in the next study.

## Study 3

Study 3 aimed to test whether the findings of Studies 1 and 2 could be extended to a non-academic achievement situation, namely work. We measured values and four goal orientations among employees.

### Methods

#### Participants and Procedure

One hundred and eighty-two employees from Israel (*M_age_* = 30.48, *SD* = 11.10, 80.2% females) participated in this study. Participants reported their values and achievement goal orientations online. All participants logged on to a website that enables, among other things, to fill in personality questionnaires. Participants completed the two questionnaires in no particular order and under no particular time constraints. All participants who reported both their values and their goal orientations during the weeks that the questionnaires were available are included in the sample. Participants did not report their jobs.

#### Instruments

##### Values

Values were measured with the 44-item version of the SVS (Schwartz, 1992) as in Studies 1 and 2. Means and scale reliabilities are presented in Table 1 and were largely similar to the findings of previous research (see Schwartz, 2005).

##### Achievement goal orientations

Three achievement goal orientations were measured using Vandewalle’s (1997) work goal orientation questionnaire. Eight amity work goal-orientation items (Levontin and Bardi, 2018, see Appendix 1) were added to the questionnaire to create one questionnaire with 21 items. Participants indicated on a scale from 1 (not at all true of me) to 7 (very much true of me) the extent to which they are motivated at work by performance-approach (termed “proving goals,” four items, α = 0.86; e.g., “I prefer to work on projects where I can prove my ability to others”), mastery (five items, α = 0.87; e.g., “I am willing to select a challenging work assignment that I can learn a lot from”), performance-avoidance (four items, α = 0.86; e.g., “I prefer to avoid situations at work where I might perform poorly”), and amity (eight items, α = 0.83; e.g., “It is important to me to assist my coworkers to succeed in their assignments”) goal orientations. As in Studies 1 and 2, before building goal orientation and value scales, each person’s responses were centered on his or her mean.

### Results and Discussion

As in Studies 1 and 2, the results of the current study show that amity goal orientation is important to employees (*M* = 5.32, *SD* = 1.00), more than performance-avoidance goal orientation [*M* = 4.07, *SD* = 1.40, *t*_(181)_ = 9.76, *p* < 0.001], but less than mastery goal orientation [*M* = 5.88, *SD* = 0.99, *t*_(181)_ = -6.38, *p* < 0.001] and less than performance-approach goal orientation [*M* = 5.59, *SD* = 1.17, *t*_(181)_ = -2.48, *p* = 0.014].

The correlations between values and goal orientations are presented in Table 2. Since we tested our four hypotheses, a correlation with a *p*-value smaller than 0.0063 would support the hypotheses, correcting for possible alpha error inflation. As expected, mastery goal orientation is positively related to self-direction values (*r* = 0.26, *p* < 0.001). The correlation between amity goal orientation and benevolence values is in the expected direction (*r* = 0.19, *p* = 0.009). The correlations between performance-approach goal orientation and power values (*r* = 0.15, *p* = 0.051), and between performance-avoidance goal orientation and security values (*r* = 0.04) were not significant.

**FIGURE 7:**
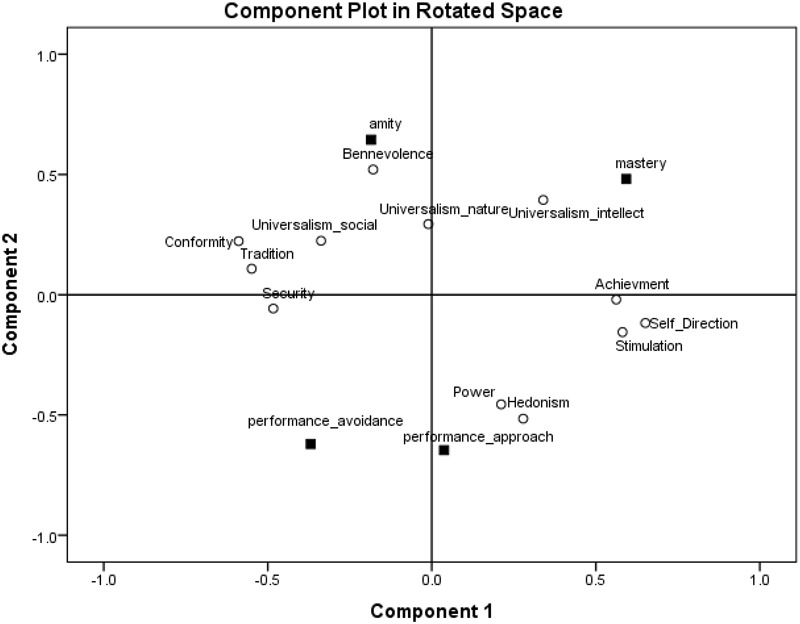
Component loadings of goal orientations and values in a two-dimensional space, Study 3.

To find where goal orientations are located in the two-dimensional values space, we ran a principal component analysis on the four goal orientations and the 12 values. In this study, unlike the universal pattern of inter-correlations among values and unlike what was found in Studies 1 and 2, achievement values were not positively correlated with power values (*r* = 0.08, *NS*) nor with hedonism values (*r* = -0.04, *NS*), but rather with self-direction values (*r* = 0.26, *p* < 0.01) suggesting that they do not have a self-enhancing motivation but rather an openness motivation in this sample.^2^ The Varimax-rotated component loadings are presented in Figure 7. As expected, amity goal orientation emerged in the area of self-transcendence values close to benevolence values. Performance-approach goal orientation emerged in the area of self-enhancement values close to power and hedonism values. Mastery goal orientation emerged in the area of openness values close to universalism-intellect values. Finally, performance-avoidance goal orientation emerged in the area of conservation values, relatively close to security values.

**FIGURE 8:**
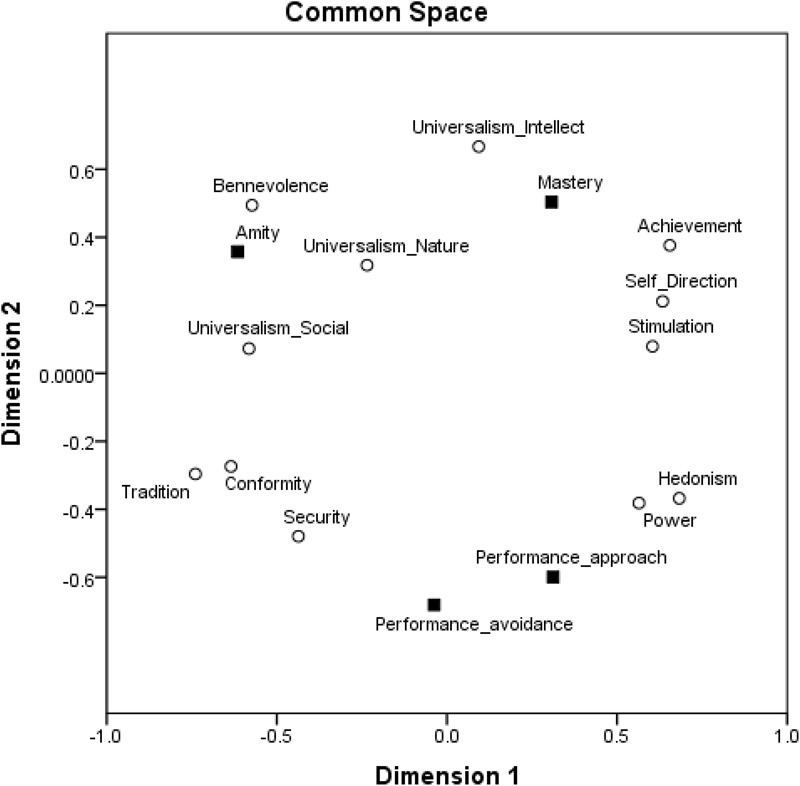
MDS of achievement goal orientations and values in a two-dimensional space, Study 3.

To further asses the structure of relations among work goal orientations and values and their locations around the circular motivational continuum, we followed a confirmatory procedure and ran MDS analyses as in Studies 1 and 2. We assessed whether the 12 values and four goal orientations form structures comparable to the structure obtained by the exploratory analysis using MDS. We used the SPSS MDS Proxscal analysis with Euclidian distance measures and the inter-correlations among the centered variables as the data. We specified an ordinal MDS and Torgerson initial configuration. Results are presented in Figure 8. The similarity of the results to those obtained by exploratory factor analysis is once more striking. Furthermore, this result is a good representation of the data: Normalized raw stress = 0.028, Stress-I = 0.167, Stress-II = 0.430, S-Stress = 0.059, DAF (dispersion accounted for) = 0.972, Tucker’s coefficient of congruence = 0.986. Specifically, as expected, amity goal orientation emerged in the area of self-transcendence values close to benevolence and universalism-social values; performance-approach goal orientation emerged in the area of self-enhancement values close to power and hedonism values; mastery goal orientation emerged in the area of openness values close to universalism-intellect, stimulation, and self-direction values, and also close to achievement values (which seemed to have an openness motivation in this sample); and performance-avoidance goal orientation emerged in the area of conservation values relatively close to security, conformity, and tradition values.

The results of this study suggest that amity goal orientation is more important to employees than performance-avoidance goal orientation, and that amity goal orientation is related to benevolence values. The results further extend the achievement goal orientations approach to include personal values as possible antecedents of work goal orientations. That is, personal values are meaningfully related to achievement goal orientations, not only in the academic context but also in the work context.

The results of Studies 1–3 suggest that in achievement situations, in support of H1, amity goal orientation is associated with benevolence values. H2 received some support since performance-approach goal orientation was associated with power values in Studies 1 and 2 but only a marginally significant correlation was found in Study 3. H3 was supported as mastery goal orientation was associated with self-direction values in all three samples. Finally, H4 was not supported as in Study 2 performance-avoidance goal orientation was associated with security values, only a marginally significant relation was found in Study 1 and no relation in Study 3.

## Mini Meta-Analysis

Having three studies enabled us to meta-analyze the correlations between values and achievement goal orientations and to provide the most reliable pattern of relations between goal orientations and values which is not dependent on specific goal orientation measure or specific achievement context. Data analysis was performed based on Hunter and Schmidt’s (1990) psychometric meta-analysis technique, to compute sample-weighted correlations. As a first step, we corrected unreliabilities for both values and goals. Then we used “comprehensive meta-analysis” software version 3^3^ to calculate the sample-weighted mean correlations (see Tables 3–6).

**Table 3:** Meta-analyses of the correlations between values and amity goal orientation, *K* = 3; *N* = 680.

Value type	Effect size			Test of null (two-tailed)		Heterogeneity		
	*p*	95% CI lower limit	95% CI upper limit	*Z*	*p*	*Q*-value	*p*	*I*^2^
Power	-0.305	-0.379	-0.243	-8.371	0.000	3.659	0.161	45.336
Achievement	-0.199	-0.271	-0.126	-5.235	0.000	0.645	0.725	0.000
Hedonism	-0.161	-0.234	-0.086	-4.203	0.000	1.395	0.498	0.000
Stimulation	-0.058	-0.132	0.018	-1.492	0.136	2.322	0.313	13.884
Self-direction	-0.222	-0.293	-0.149	-5.861	0.000	6.060	0.048	66.996
Universalism-intellect	-0.104	-0.178	-0.028	-2.696	0.007	4.560	0.102	56.141
Universalism-nature	0.160	0.086	0.233	4.181	0.000	0.000	1.000	0.000
Universalism-social	0.297	0.227	0.364	7.934	0.000	4.248	0.120	52.916
Benevolence	**0**.**342**	**0**.**274**	**0**.**407**	**9**.**230**	**0**.**000**	3.896	0.143	48.670
Tradition	0.180	0.106	0.252	4.719	0.000	0.686	0.710	0.000
Conformity	-0.018	-0.204	0.169	-0.337	0.736	11.353	0.003	82.383
Security	-0.130	-0.203	-0.055	-3.377	0.001	5.913	0.052	66.179

*Hypothesized correlations are in bold; CI = confidence interval. *I*^2^ = % variability due to heterogeneity (0–100).*

**Table 4:** Meta-analyses of the correlations between values and performance-approach goal orientation, *K* = 3; *N* = 680.

Value type	Effect size			Test of null (two-tailed)		Heterogeneity		
	*p*	95% CI lower limit	95% CI upper limit	*Z*	*p*	*Q*-value	*p*	*I*^2^
Power	**0**.**347**	**0**.**279**	**0**.**412**	**9**.**382**	**0**.**000**	6.222	0.045	67.854
Achievement	0.221	0.148	0.292	5.831	0.000	9.245	0.010	78.367
Hedonism	0.157	0.082	0.230	4.101	0.000	1.528	0.466	0.000
Stimulation	0.002	-0.073	0.078	0.061	0.951	2.024	0.364	1.172
Self-direction	0.106	0.031	0.180	2.751	0.006	2.503	0.286	10.081
Universalism-intellect	-0.015	-0.090	0.061	-0.380	0.704	9.988	0.007	79.977
Universalism-nature	-0.093	-0.167	-0.017	-2.412	0.016	10.466	0.005	80.890
Universalism-social	-0.181	-0.253	-0.107	-4.737	0.000	0.701	0.704	0.000
Benevolence	-0.300	-0.367	-0.230	-8.020	0.000	3.515	0.172	43.101
Tradition	-0.216	-0.287	-0.143	-5.691	0.000	2.642	0.267	24.311
Conformity	-0.071	-0.146	0.004	-1.851	0.064	1.253	0.534	0.000
Security	0.115	0.040	0.189	3.004	0.003	1.879	0.391	0.000

*Hypothesized correlations are in bold; CI = confidence interval. *I*^2^ = % variability due to heterogeneity (0–100).*

**Table 5:** Meta-analyses of the correlations between values and mastery goal orientation, *K* = 3; *N* = 680.

Value type	Effect size			Test of null (two-tailed)		Heterogeneity		
	*p*	95% CI lower limit	95% CI upper limit	*Z*	*p*	*Q*-value	*p*	*I*^2^
Power	-0.098	-0.172	-0.023	-2.551	0.011	4.072	0.131	50.882
Achievement	0.192	0.118	0.264	5.040	0.000	11.507	0.003	82.620
Hedonism	-0.202	-0.273	-0.128	-5.293	0.000	1.600	0.449	0.000
Stimulation	0.010	-0.065	0.086	0.269	0.788	17.692	0.000	88.695
Self-direction	**0**.**262**	**0**.**190**	**0**.**380**	**6**.**947**	**0**.**000**	4.277	0.118	53.237
Universalism-intellect	0.177	0.103	0.249	4.635	0.000	17.560	0.000	88.610
Universalism-nature	0.058	-0.018	0.133	1.498	0.134	1.389	0.499	0.000
Universalism-social	-0.112	-0.186	-0.037	-2.907	0.004	6.383	0.041	68.665
Benevolence	0.123	0.048	0.197	3.200	0.001	3.132	0.209	36.140
Tradition	-0.073	-0.147	0.003	-1.888	0.059	7.710	0.021	74.059
Conformity	0.049	-0.027	0.124	1.263	0.207	6.212	0.045	67.804
Security	-0.146	-0.219	-0.071	-3.814	0.000	2.415	0.299	17.197

*Hypothesized correlations are in bold; CI = confidence interval. *I*^2^ = % variability due to heterogeneity (0–100).*

**Table 6:** Meta-analyses of the correlations between values and performance-avoidance goal orientation, *K* = 3; *N* = 680.

Value type	Effect size			Test of null (two-tailed)		Heterogeneity		
	*p*	95% CI lower limit	95% CI upper limit	*Z*	*p*	*Q*-value	*p*	*I*^2^
Power	0.282	0.211	0.350	7.499	0.000	15.205	0.000	86.847
Achievement	-0.042	-0.117	0.034	-1.083	0.279	6.973	0.031	71.317
Hedonism	0.166	0.092	0.239	4.351	0.000	0.108	0.948	0.000
Stimulation	-0.082	-0.157	-0.007	-2.132	0.033	0.830	0.660	0.000
Self-direction	-0.044	-0.119	0.032	-1.130	0.258	0.102	0.950	0.000
Universalism-intellect	-0.283	-0.351	-0.212	-7.533	0.000	0.615	0.735	0.000
Universalism-nature	-0.131	-0.205	-0.056	-3.421	0.001	1.941	0.379	0.000
Universalism-social	-0.096	-0.170	-0.020	-2.482	0.013	4.545	0.103	56.000
Benevolence	-0.253	-0.322	-0.181	-6.688	0.000	3.999	0.135	49.993
Tradition	0.027	-0.049	0.102	0.696	0.486	7.337	0.026	72.740
Conformity	0.109	0.034	0.183	2.846	0.004	7.124	0.028	71.926
Security	**0**.**200**	**0**.**127**	**0**.**272**	**5**.**256**	**0**.**000**	5.716	0.057	65.012

*Hypothesized correlations are in bold; CI = confidence interval. *I*^2^ = % variability due to heterogeneity (0–100).*

### Results

#### Amity Goal Orientation

Consistent with our hypothesis (see Table 3) amity goal orientation was positively related to benevolence values (r = 0.342). Amity goal orientation was also found to be positively related to universalism-social values (*p* = 0.297), universalism-nature values (*p* = 0.160), and tradition values (*p* = 0.180), all of them are adjacent to benevolence values in the values circle (see Figure 1 above).

#### Performance-Approach Goal Orientation

Consistent with our hypothesis (see Table 4) performance-approach goal orientation was positively related to power values (*p* = 0.347). Performance-approach goal orientation was also found to be positively related to achievement values (*p* = 0.221), hedonism values (*p* = 0.157), and security values (*p* = 0.115), all of them are adjacent to power values in the values circle (see Figure 1 above).

#### Mastery Goal Orientation

Consistent with our hypothesis (see Table 5), mastery goal orientation was positively related to self-direction values (*p* = 0.262). Mastery goal orientation was also found to be positively related to the adjacent values of universalism-intellect (*p* = 0.177) and benevolence (*p* = 0.123). However, mastery goal orientation was also positively related to achievement values (*p* = 0.192) that are not adjacent to self-direction values in the values circle (see Figure 1 above).

#### Performance-Avoidance Goal Orientation

Consistent with our hypothesis (see Table 6), performance-avoidance goal orientation was positively related to security values (*p* = 0.200). Performance-avoidance goal orientation was also found to be positively related to the adjacent conformity values (*p* = 0.109) and power values (*p* = 0.282). However, performance-avoidance goal orientation was also positively related to hedonism values (*p* = 0.166 that are not adjacent to security values in the values circle (see Figure 1 above).

## General Discussion

Using basic values, we found evidence that amity goal orientation is related to the prosocial motivation underlying self-transcendence values – benevolence and universalism-social values. We showed that people view amity goal orientation as relatively important in achievement situations, more than some other goal orientations, both in the academic context and in the work context.

In addition to introducing a new motivational basis for amity goal orientation, our studies demonstrated the links between other goal orientations and basic values. These links provide evidence for the motivational base of these goal orientations. Specifically, we found evidence in line with the idea that performance-approach goal orientation is related to self-enhancement values, power, achievement, and hedonism values; mastery goal orientation is related to openness values, self-direction, and universalism-intellect values and also to achievement values; and performance-avoidance goal orientation is related to the conservation values of security and conformity and also to power and hedonism values.

The two-dimensional joint structures of relations between values and goal orientations were largely consistent across three studies, achievement contexts, and methods of analysis. The pattern of relations between values and academic achievement goal orientations as reported by students (Studies 1 and 2) was very similar to the pattern of relations between values and work achievement goal orientations as reported by employees (Study 3). Furthermore, the structure of relations between values and achievement goal orientations replicated across methods of analysis (factor analysis and MDS), implying that researchers can use the more familiar and accessible method of factor analysis to examine structures that are based on two orthogonal dimensions.

The results of the current research provide some explanation for the relations found in the literature among goal orientations and advance our understanding of their nature, underlying motivation, and their possibility of co-occurring. The positive correlations often found in the literature between performance-approach and mastery goal orientations imply that people often hold them simultaneously (e.g., Darnon et al., 2010). Indeed, meta-analyses resulted in a positive correlation between mastery and performance-approach goal orientations (*k* = 193 studies, ρ = 0.19, Hulleman et al., 2010; *k* = 148 studies, ρ = 0.15, Payne et al., 2007). Furthermore, achievement settings such as academic institutions tend to encourage both mastery and performance-approach goal orientations (Darnon et al., 2009). The results of the current research suggest that people hold both mastery and performance-approach goal orientations because they are based on the non-conflicting motivations of openness to change and self-enhancement, and are both related to achievement values. Similarly, performance-approach and performance-avoidance goal orientations are often found to be highly correlated (e.g., Darnon et al., 2007). Indeed, meta-analyses resulted in a strong positive correlation between performance-approach and performance-avoidance goal orientations (*k* = 147 studies, ρ = 0.40, Hulleman et al., 2010; *k* = 48 studies, ρ = 0.40, Payne et al., 2007), and some researchers even treat them as the same type of goal orientation (e.g., Duda, 2005; Urdan and Mestas, 2006; Bong, 2009). The results of the current research suggest that people may hold both performance-approach and performance-avoidance goal orientations because they are based on the non-conflicting motivations of self-enhancement and conservation, and are both related to power, hedonism, and security values. Finally, the positive correlation found between mastery and amity goal orientations (Levontin and Bardi, 2018) can also be explained by the results of the current research as they are based on the non-conflicting motivations of openness to change and self-transcendence and are both related to benevolence values.

However, mastery and performance-avoidance were found to be negatively correlated in some achievement situations (e.g., VandeWalle, 1997) and not correlated in others (e.g., Elliot and McGregor, 2001). Meta-analyses yielded either no correlation between mastery and performance-avoidance goal orientations (*k* = 141 studies, ρ = -0.01, Hulleman et al., 2010), or a negative correlation between them (*k* = 48 studies, ρ = -0.23, Payne et al., 2007). The results of the current research suggest that people usually would not hold both high mastery and high performance-avoidance goal orientations because they are based on the conflicting motivations of openness to change and conservation. We found that when analyzed with values, mastery and performance-avoidance goal orientations emerge in two opposites of the values and goal orientations space

Importantly, we found that performance-approach goal orientation emerged opposite to amity goal orientation. This structure suggests that these two goal orientations may reflect the interpersonal dimension of goal orientations of competition (in performance-approach) and cooperation (in amity). Future research should study further the antecedents and consequences of self-focused and other-focused goal orientations. Specifically, it seems that mastery goal orientation is a self-focused goal orientation focused at competence improvement as reflected in self-direction values, whereas performance-avoidance goal orientation is a self-focused goal orientation focused at the avoidance of revealing low competence as reflected in security values. Performance-approach goal orientation is an other-focused goal orientation focused at competition, as reflected by power values, while amity goal orientation is an other-focused goal orientation focused on cooperation as reflected by benevolence values.

Future research could also attempt to incorporate the approach/avoidance dimension into amity goal orientation. We expect the same pattern of relations with values, whether we measure amity-approach (e.g., “It is important for me to cooperate with others at school”) or amity-avoidance (e.g., “It is important for me to avoid non-cooperation with others at school”). However, there may be merit in examining other aspects of amity approach versus avoidance, such as consequences in terms of well-being or satisfaction, as previous research generally finds that approach orientations tend to be positively associated with well-being and satisfaction whereas avoidance orientations tend to be positively associated with anxiety and depression (Elliot and McGregor, 2001).

## Conclusion

To conclude, in this paper, we have revealed a systematic pattern of relations between personal values (broad motivations) and achievement goal orientations (context-specific goal orientations). This comparison allowed revealing the values related to amity achievement goal orientation, as well as the motivational correlates of other goal orientations. We hope that this paper will stimulate further research that combines amity achievement goal orientation in order to enhance our understanding of motivation, emotion, and performance in achievement situations.

## Ethics Statement

This study was carried out in accordance with the recommendations of institutional RB committee with written informed consent from all subjects. All subjects gave written informed consent. The protocol was approved by the institutional IRB committee.

## Author Contributions

Both LL and AB substantially contributed to the conception and design of the work, interpreted the data, approved the version to be published, and agreed to be accountable for all aspects of the work in ensuring that questions related to the accuracy or integrity of any part of the work are appropriately investigated and resolved. LL led data analysis and drafted the work. AB revised it critically for important intellectual content.

## Conflict of Interest Statement

The authors declare that the research was conducted in the absence of any commercial or financial relationships that could be construed as a potential conflict of interest.

## Footnotes

1 Mastery-avoidance goal orientation is likely to have the same pattern of correlations with values as mastery-approach goal orientation. We opted to measure mastery-approach goal orientation for two reasons. First, because openness to change values are naturally more about approaching goals than about avoiding goals (see Woltin and Bardi, 2018). Second, because mastery-approach has been established in much more research than mastery-avoidance (Hulleman et al., 2010).

2 The structure of the 10 values sometimes deviates from the theoretical structure. Deviations from the theoretical structure are reported in Schwartz (1992). Similar to our findings, in Table V of that paper it is reported that in five out of 40 samples, achievement was not compatible with power, and in seven samples, achievement was not compatible with hedonism.

3 https://www.meta-analysis.com/

## Appendix 1

Academic amity goal orientation items (Study 1)

1. It is important for me to cooperate with others at school.
2. It is important for me to assist my friends to succeed in school.
3. One of my goals at school is to help others to succeed.
4. It is important to me that my friends will succeed as well as I do.

Work amity goal orientation items (Study 3)

1. It is important for me to cooperate with my coworkers.
2. It is important for me to work together with my close friends.
3. I think it is important to cooperate with others at work.
4. I believe that my coworkers and I have similar high abilities.
5. It is important for me to assist my coworkers to succeed in their assignments.
6. To be honest, I prefer working with others than working alone.
7. It is important to me that my best friends at work will do as well as I do.
8. An assignment that requires cooperation with others is more enjoyable for me.
